# Plasma Biomarker for Post-concussive Syndrome: A Pilot Study Using an Alternating Current Electro-Kinetic Platform

**DOI:** 10.3389/fneur.2020.00685

**Published:** 2020-07-14

**Authors:** Jean M. Lewis, Sanjay Dhawan, Augustine C. Obirieze, Benjamin Sarno, Johnny Akers, Michael J. Heller, Clark C. Chen

**Affiliations:** ^1^Department of Nanoengineering, University of California, San Diego, San Diego, CA, United States; ^2^Department of Neurosurgery, University of Minnesota, Minneapolis, MN, United States; ^3^VisiCELL Medical Inc., San Diego, CA, United States; ^4^Department of Bioengineering, University of California, San Diego, San Diego, CA, United States

**Keywords:** alternating current electrokinetics, traumatic brain injury, biomarkers, extracellular vesicles, concussion

## Abstract

**Background:** Technology platforms that afford biomarker discovery in patients suffering from traumatic brain injury (TBI) remain an unmet medical need. Here, we describe an observational pilot study to explore the utility of an alternating current electrokinetic (ACE) microchip device in this context.

**Methods:** Blood samples were collected from participating subjects with and without minor TBI. Plasma levels of glial fibrillary acidic protein (GFAP), Tau, ubiquitin C-terminal hydrolase L1 (UCH-L1), and cell-free DNA (cfDNA) were determined in subjects with and without minor TBI using ACE microchip device followed by on-chip immunofluorescent analysis. Post-concussive symptoms were assessed using the Rivermead Post Concussion Symptoms Questionnaire (RPCSQ) at one-month follow-up.

**Results:** Highest levels of GFAP, UCH-L1, and Tau were seen in two minor TBI subjects with abnormality on head computed tomography (CT). In patients without abnormal head CT, Tau and GFAP levels discriminated between plasma from minor-TBI and non-TBI patients, with sensitivity and specificity of 64–72 and 50%, respectively. Plasma GFAP, UCH-L1, and Tau strongly correlated with the cumulative RPCSQ score. Plasma UCH-L1 and GFAP exhibited highest correlation to sensitivity to noise and light (*r* = 0.96 and 0.91, respectively, *p* < 0.001). Plasma UCH-L1 and Tau showed highest correlation with headache (*r* = 0.74 and 0.78, respectively, *p* < 0.001), sleep disturbance (*r* = 0.69 and 0.84, respectively, *p* < 0.001), and cognitive symptoms, including forgetfulness (*r* = 0.76 and 0.74, respectively, *p* < 0.001), poor concentration (*r* = 0.68 and 0.76, respectively, *p* < 0.001), and time required for information processing (*r* = 0.77 and 0.81, respectively, *p* < 0.001). cfDNA exhibited a strong correlation with depression (*r* = 0.79, *p* < 0.01) and dizziness (*r* = 0.69, *p* < 0.01). While cfDNA demonstrated positive correlation with dizziness and depression (*r* = 0.69 and 0.79, respectively, *p* < 0.001), no significant correlation was observed between cumulative RPCSQ and cfDNA (*r* = 0.07, *p* = 0.81).

**Conclusion:** We provide proof-of-principle results supporting the utility of ACE microchip for plasma biomarker analysis in patients with minor TBI.

## Introduction

Each year, over 1.7 million people in the U.S. suffer traumatic brain injury (TBI), requiring medical attention (1). Depending on the severity of the clinical presentation, TBI is classified into mild, moderate, or severe (2). >80% of head trauma patients who present to the emergency room suffer from mild TBI. While the majority of these mild TBI patients are discharged from the emergency room on the same day and recover without detectable long-term sequelae, 30% of mild TBI patients will have persistent “post-concussive” symptoms that significantly compromise their quality of life, including headache, fatigue, as well as altered sensation, sleep, and attention span (3–5). Notably, ~8% of mild TBI patients demonstrate visible injury to the cerebrum on computerized tomography (CT) (6). Direct and indirect costs associated medical care and productivity loss associated with mild TBI exceeds $60 billion annually (7).

Mild TBI involves complex pathophysiologic processes associated with microscopic shearing of cells in the central nervous system secondary to traumatic biomechanical forces to the head (8). Such shearing induces damage to the cell, resulting in the release of neuronal and astrocytic proteins or cell-free DNA (cfDNA) not normally found in the extracellular space (9), including glial fibrillary acidic protein (GFAP) (10), ubiquitin carboxyl-terminal hydrolase L1 (UCH-L1) (11), and Tau (12). Tau is a microtubule stabilizing protein abundant in neurons (13), and released into the extracellular space upon neuronal damage (14). GFAP is an intermediate filament protein highly abundant in astrocytes (15), and increased levels in blood or CSF are linked to axonal injury (16, 17). UCH-L1 is a neuronal protease, with increased levels linked to brain injury (18–20). Since ~20% of circulating blood volume perfuses the cerebrum and TBI often compromises the blood-brain barrier, these released proteins can be detected in peripheral blood drawn from TBI patients (21). The high sensitivity and specificity of GFAP and UCH-L1 as proxy for abnormal CT following TBI have paved way to clearance by the U.S. Food and Drug Administration (FDA) for their use in TBI work-up. While cfDNA has been shown to predict mortality in severe TBI patients, it becomes imperative to explore the utility of cfDNA in the management of mild TBI patients that constitute ~ 75% of the patients with TBI annually (22–24).

In contrast to the number of tools that afford study and characterization of TBI associated structural injuries, there are currently no standardized and well-established clinical criteria or biomarkers that afford identification of the minority of mild TBI patients who suffer long-term sequelae despite absence of detectable structural damage to the cerebrum (5, 25). Currently, study of these symptoms relies on questionnaires, such as the Rivermead Post Concussion Symptoms Questionnaire (RPCSQ) (26, 27), that are administered at regular intervals after the injury. Development of predictive tools for likelihood of post-concussive symptoms can meaningfully impact the care of mild TBI patients and advance our understanding of the underlying pathophysiology. Here, we explore the utility of plasma GFAP, UCH-L1, Tau, and cell-free DNA in this context using an alternating current electro kinetic (ACE) microchip device.

**Figure 1:**
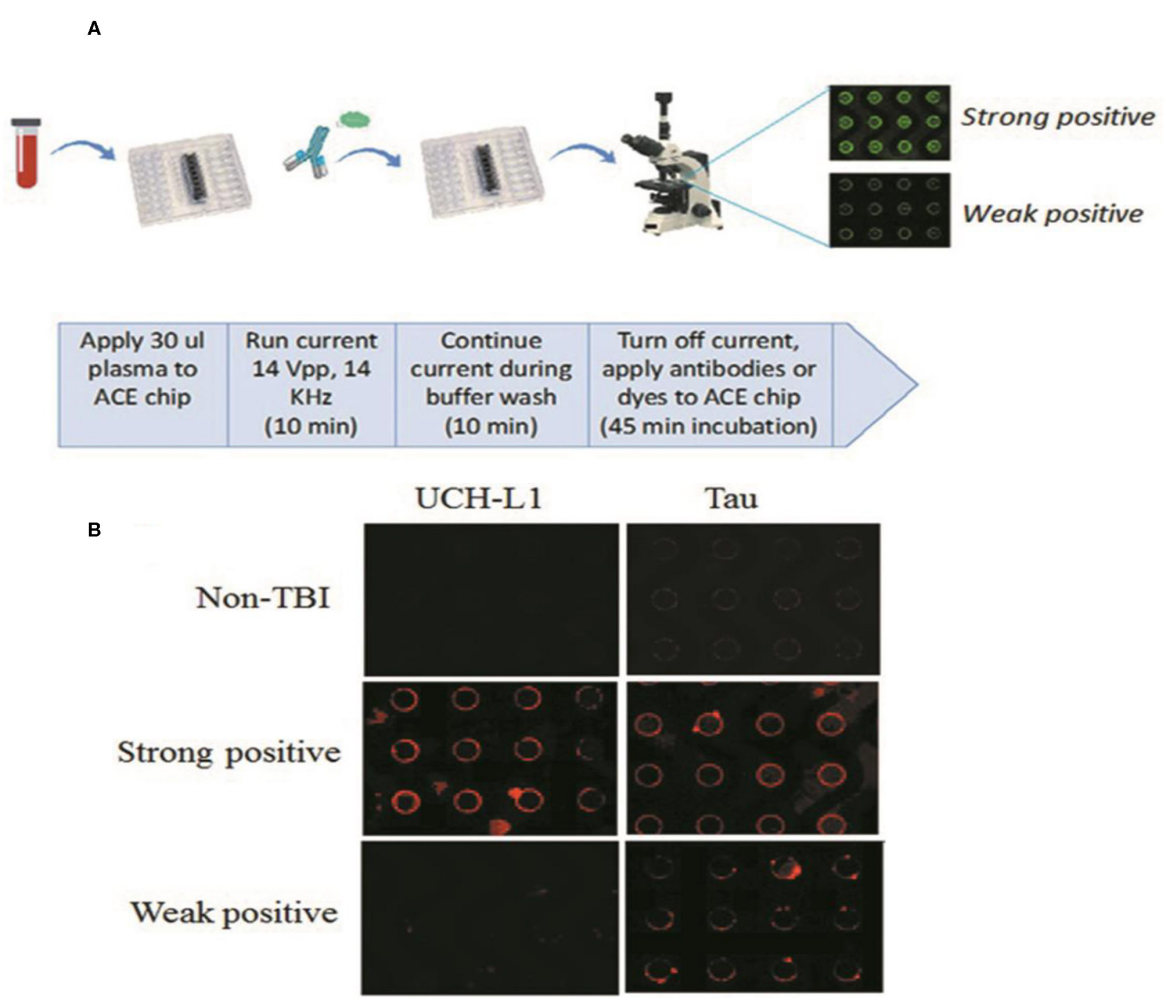
**(A)** Overview of ACE microchip immunoassay workflow. Shown is a fluidics cartridge containing the 14 × 52 mm-sized ACE chip configured with eight sample chambers. When current is applied, extracellular vesicles (EVs), and other nanoparticles are drawn to the edges of the circular electrodes; the buffer wash serves to remove larger unbound cellular debris and smaller soluble plasma components from the chip. The current is then turned off, mixtures of fluorescent antibodies or dyes selective for each biomarker are added, and the concentration of biomarker proteins around the electrodes can be visualized as circular patterns of fluorescence. Image analysis provides a quantitative comparison of fluorescence intensities. **(B)** Representative examples showing relative abundance of UCH-L1 and Tau using the ACE microarray and on-chip immune-fluorescence (IF) analysis. ACE, alternating current electrokinetic. UCH-L1, ubiquitin C-terminal hydrolase L1.

ACE microarray chips use alternating current to isolate macro-molecular complexes from bio-fluids, such as blood or cerebrospinal fluid, in a highly efficient manner (28). Of note, nearly all plasma GFAP, Tau, and UCH-L1 are found in these macro-molecular complexes (29, 30). The chip-retained protein can be labeled with fluorescent antibodies or dyes specific for the biomarker of interest. Quantitative on-chip fluorescent imaging analysis is then carried out Figure 1A (31, 32). In this pilot study, we correlated the levels of ACE isolated GFAP, UCH-L1, Tau and cell-free DNA from blood drawn at the time of injury to Rivermead questionnaire results collected at one-month after head injury from mild TBI patients.

## Methods

### Study Design

Research protocol was approved by the University of California San Diego Institutional Review Board (IRB #120345X). Study subjects were patients who sought care at the Emergency Department of the University of California San Diego Hillcrest Medical Center. The hospital serves the endogenous population in the San Diego County. Inclusion criteria include: patients age > 18 and capable for consent based on- (i) determination of the treating physicians, and (ii) the University of California San Diego Brief Instrument for Assessing Decisional Capacity for Clinical Research (UBACC) 10 item scale administered by the trained clinical coordinator (33). There were no explicit exclusion criteria.

To avoid study interference with the standard-of-care for patients in the emergency ward, the study is designed such that the study coordinator regularly checks in with the treating physician to identify potential candidate for consent. Treatment decisions, including indication for head CT, were made entirely by the treating physician.

Each patient underwent blood draw as per standard-of-care. Informed consent was obtained from each participating patient by a dedicated research assistant on the day of presentation. Blood samples that remain after standard laboratory chemistry were collected, and no blood draw beyond the standard-of-care venipuncture was performed. The residual blood was collected from the chemistry lab the day after the presentation after adequate laboratory values were reported to the Electronic Medical Record. Hemolyzed samples were not collected. The volume of the residual blood ranged from 100 μl to 1.5 ml. These samples were collected within 72 h of blood draw and stored in liquid nitrogen.

Samples were collected from consecutive patients who presented with history of minor TBI, defined by Glasgow Coma Scale (GCS) of > 13, and without history of loss of consciousness (34, 35). The collection period extended between 2015 and 2016. In parallel, our protocol allowed collection of blood from patients who presented to the Emergency Ward with non-TBI and non-neurologic complaints. Patients who required major medical intervention, such as cardiac catheterization or surgical intervention were excluded from the study to minimize risk of adding stress of study participation to the patient. Residual blood after completion of standard chemistry was collected from these patients in the same manner as described above. Samples were stored in 4C refrigeration before transportation to the biorepository. After transportation to the biorepository, the samples were de-identified and stored as processed plasma by centrifugation at 1,300 rpm for 10 min followed by 3,000 rpm for 10 min. Samples were stored at −80C before analysis. Procedures for de-identification and human subject protection were performed in compliance to the hospital policy. The staff who analyzed the sample was blinded to the clinical history of the patients.

For all study subjects, participation in this study did not alter the standard of care, including the routine one-month post-presentation follow-up for patients with minor TBI. For the patients who presented with minor TBI, they underwent standard-of-care work-up as determined by the attending Emergency Ward faculty physician, including non-contrast computed tomography (CT) scan of the head. The only exception to the above is that the Rivermead Post Concussion Symptoms Questionnaire (RPCSQ) was administered by a trained study coordinator at this one-month follow-up (26, 27). During this assessment, the patients are asked to report only symptoms that were not present prior to the minor TBI.

### ACE-Based Processing of Plasma Samples

ACE chips were purchased from Biological Dynamics, Inc. (San Diego, CA). ACE-based processing of plasma samples has been demonstrated previously (36). A syringe pump set to withdrawal mode served to regulate fluid flow across the ACE chip. Tygon tubing (inner diameter, 0.020 inches; outer diameter, 0.060 inches) was attached with superglue to either end of the chip, both ends were capped with syringe needles, and a 1 ml syringe was attached to one end. Twenty-five μl of thawed plasma was drawn onto the chip. An alternating current (AC) electric field was applied to the chip for 10 min at 14 volts peak-to-peak and 15 kHz to immobilize extracellular vesicles and other nanoparticles onto the microelectrode edges. The technique to isolate exosomes from plasma samples has been demonstrated previously (28). With the AC field still on, the ACE chip was then washed with 200 μl of 0.5X PBS for an additional 10 min. The time taken for the entire process was 20 min for the ACE-based isolation, plus an additional 45–90 min for antibody binding steps (Figure 1A).

### On-Chip Immunofluorescent Analysis

Two biomarkers were tested simultaneously on each chip, using FITC and TRITC filter sets on the microscope. On-chip immunofluorescent analysis has been demonstrated previously (36). The manufacturer and catalog number of the antibodies used are as follows: Rabbit anti-UCH-L1: Cell Signaling Technologies, clone D3T2E, #13179, diluted 1:800; Alexa Fluor 594-goat anti rabbit IgG, highly cross-adsorbed, Life Technologies #A11039, diluted 1:2000; Alexa Fluor 488 mouse anti GFAP: clone 1B4, BD Biosciences #560297, diluted 1:10; Mouse anti-Tau: clone TAU-5 (total-tau), Life Technologies #ABH0042, diluted 1:50; Alexa Fluor 594-goat anti mouse IgG, highly cross-adsorbed, Life Technologies #A11032, diluted 1:2000.

To enable access of the antibodies to proteins within the luminal space of the vesicles, EV membranes were permeabilized using 0.1% saponin for 10 min. To label cfDNA, the selective dye YOYO-1 was added to a concentration of 1:5,000. Antibody incubations were performed for 45–90 min at room temperature, or, if recommended by the manufacturer, overnight at 4°C for optimal binding. For directly conjugated Alexa Fluor 488-anti-GFAP antibody (BD Pharmingen), samples were washed with PBS, then visualized and photographed for further analysis. For anti-Tau or anti-UCH-L1 (Life Technologies; Cell Signaling Technology), following the wash, Alexa Fluor 594-conjugated secondary antibody (Novex, Life Technologies) was incubated for an additional 60 min at room temperature. Following an additional wash, samples were viewed on the microarray chips using an Olympus BX51W epifluorescence microscope with a 4X objective and imaged with Olympus software. All image acquisition parameters were the same for the same fluorophore.

To quantify relative levels of fluorescent antibody-labeled Tau, GFAP, UCH-L1, and cfDNA for each sample, photographic images of each ACE-chip were imported to ImageJ (“FIJI”; National Institutes of Health). A circle was drawn around each of eight electrodes, and pixels measured. Background subtracted was the minimum number of pixels measured for each electrode, and averages and standard deviations were calculated. Direct 3D representations of the images were created using the “3D interactive viewer” plug-in for ImageJ.

Figure 1B shows representative examples of plasma sample analyses from the study cohort for the relative abundance of UCH-L1 and Tau.

### Statistical Analysis

Models were used to predict the severity of injury with the probability of intracranial abnormality post TBI. The probability threshold was chosen as that which minimizes the Euclidean distance from point (0.1), or the upper-left corner, on the receiver operating characteristic (ROC) curve. ROC curve were calculated to determine the area under the curve (ROC-AUC) for different biomarkers. Based on the ROC-AUC, the optimal rIF values for discriminating minor-TBI plasma from non-TBI plasma were calculated for different biomarkers.

Model predictions were compared to observed diagnoses and performance metrics were calculated, including sensitivity, specificity, positive predictive value (PPV), negative predictive value (NPV), and the area under the ROC curve (AUC). Heat maps were constructed and Pearson correlation coefficients were calculated between GFAP, UCH-L1, Tau, cfDNA, and cumulative RPCSQ score. Correlation analysis between GFAP, UCH-L1, Tau, cfDNA, and individual RPCSQ symptoms was also performed. All analyses were carried out using open-source statistical analysis software (R version 3.5.0).

## Results

### Demographics and Clinical Course of the Study Cohort

**Figure 2:**
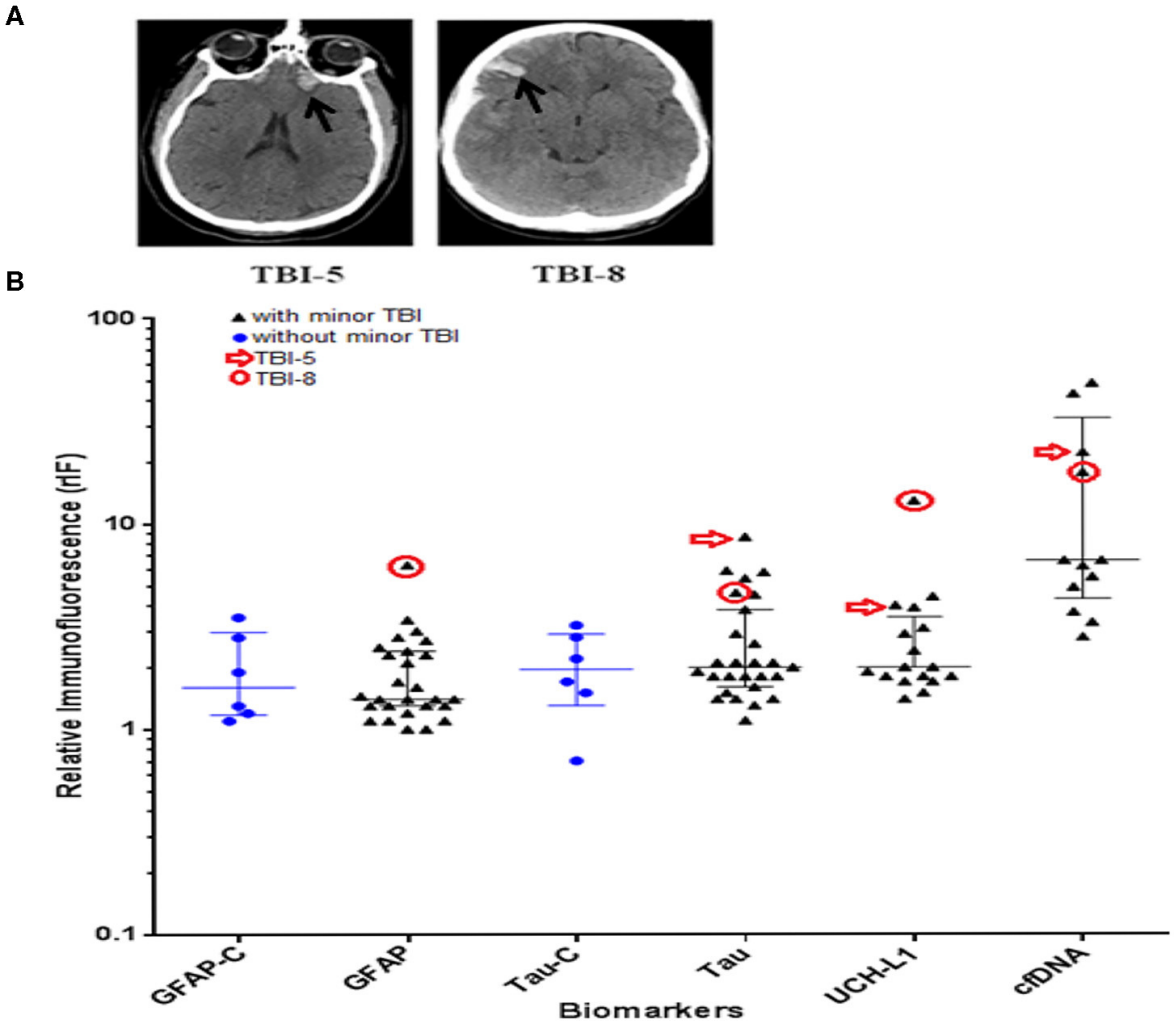
**(A)** CT scan images showing contusion in patient #5 and #8 with minor traumatic brain injury (TBI) (black arrow). **(B)** Scattered dot plots illustrating distribution of relative immunofluorescence (rIF) (median with inter-quartile range) values for GFAP, Tau, UCH-L1, and cfDNA in TBI cohort, and GFAP and Tau in non-TBI cohort (represented as GFAP-C and Tau-C, respectively). Blue dots represent the rIF values for Tau and GFAP in patients with no history of head trauma. Black triangles represent the rIF values for Tau, GFAP, UCH-L1, and cfDNA in patients with history of head trauma. rIF values for patient #5 are denoted by red arrow; rIF values for patient #8 are encircled in red. GFAP, glial fibrillary acidic protein; UCH-L1, ubiquitin C-terminal hydrolase L1; cfDNA, cell-free DNA.

The study enrolled 27 minor TBI subjects and 6 non-TBI subjects between December of 2015 and March of 2016. The demographic of the study population is as indicated in Table 1. The mean age of the minor-TBI and non-TBI cohort was 58.5 ± 16.4 and 34.6 ± 10.6 years, respectively. The male to female ratio were approximately 1.7:1 and 1:1 for the TBI and non-TBI cohort, respectively. All patients in the minor-TBI cohort underwent head CT as a part of their care. Except for the two patients (2/27 or 7%) who showed evidence of contusion on head CT (Figure 2A), all patients had negative head CTs and were discharged from the Emergency Ward on the day of the presentation. The two patients with abnormal head CT were admitted to the hospital for over-night observation. Both underwent interval surveillance imaging demonstrating stability of CT finding before discharge on the following day. The non-TBI patients presented with chest or abdominal discomfort or lower extremity pain. None of the patients in the non-TBI cohort underwent head CT. Diagnostic work-up were unremarkable and the patients were discharged on the day of intervention.

**Table 1:** Summary statistics of demographics of the study population with comparative analysis in minor traumatic brain injury (TBI) and non-TBI patients.

**Variable**	**Minor TBI cohort (*n* = 27)**	**Non-TBI cohort (*n* = 6)**	***p*-value**
Gender, *n* (%)			0.65
Male	17 (63)	3 (50)	
Female	10 (37)	3 (50)	
Age (years, mean ± SD)	58.5 ± 16.4	34.6 ± 10.6	0.002
Presentation: *n*	Fall: 16	Abdominal Pain: 4	-
	Syncope: 5	Chest Pain: 1	
	MVA: 4	Lower extremity pain: 1	
	Others: 2		

*MVA, motor vehicle accident; SD, standard deviation. Others include head injury and scalp laceration. SD, standard deviation*.

### Biomarker Comparison Between Subjects With and Without Abnormal Head CT

Plasma samples from the study cohort were analyzed for the relative abundance of GFAP, Tau, UCH-L1, and cfDNA using the ACE microarray and on-chip immunofluorescence (IF) analysis. Relative immunofluorescence (rIF) level was determined for each sample. We first posed the question of whether any of the biomarkers were elevated in the two minor-TBI patients with abnormal head CT (subject 5 and subject 8) relative to all remaining patients. We performed this analysis in a qualitative manner since the sample size is too small for meaningful quantitative assessment. Consistent with the published utility of GFAP and UCH-L1 (37), plasma from subject eight showed significantly elevated levels of both proteins. In fact, this subject harbored the highest level of both GFAP and UCH-L1 for all study cohorts. Plasma from subject 5 also showed significantly elevated UCH-L1 (Figure 2B).

Notably, plasma from both subjects 5 and 8 also harbored significantly elevated Tau levels. These observations suggest the utility of plasma Tau as biomarker for structural brain injury after TBI. In contrast, the plasma level of cfDNA in subjects 5 and 8 were not significantly elevated relative to other study cohorts.

As a proof-of-principle study, these results support an association between elevated plasma GFAP, UCH-L1, and structural TBI demonstrated on head CT.

### Biomarker Comparison Between Mild-TBI and Non-TBI Subjects

We wished to determine whether any of the biomarkers studied could discriminate the plasma collected from minor-TBI patients relative to non-TBI patients without abnormal head CT. We were only able to complete this analysis for plasma GFAP and Tau. We additionally excluded subjects 5 and 8 from this analysis since we were interested to characterize the value of these biomarkers in head CT negative TBI patients. Receiver operator characteristic curve were calculated to determine the area under the curve (ROC-AUC) for GFAP and Tau. Based on the ROC-AUC, the optimal rIF values for discriminating minor-TBI plasma from non-TBI plasma was 1.75 for Tau and 1.35 for GFAP (Table 2A). Using these cut-offs, the sensitivity and specificity of discriminating plasma between minor TBI and non-TBI patients was calculated and is shown in Table 2B. In this analysis, Tau and GFAP performed similarly, with sensitivity of 72 and 64%, respectively, and specificity of 50% for both proteins. The combinations of GFAP and Tau did not significantly improve the sensitivity or specificity relative to the individual biomarker (Table 2C).

**Table 2A:** Performance metrics of GFAP and Tau biomarkers.

**Area under curve (AUC) and cut-off relative immune fluorescence (rIF) value for GFAP and Tau**.		
**Biomarker**	**AUC**	**Cut-off rIF**
Tau	0.54 (0.24–0.83)	1.75
GFAP	0.48 (0.16–0.79)	1.35

**Table 2B:** Sensitivity, Specificity, Positive predictive value (PPV) and Negative predictive value (NPV) for GFAP and Tau.

**Biomarker**	**Sensitivity**	**Specificity**	**PPV**	**NPV**
Tau	72% (0.54–0.89)	50% (0.10–0.90)	85.7% (0.70–1.00)	30% (0.01–0.58)
GFAP	64% (0.45–0.82)	50% (0.10–0.90)	84.2% (0.67–1.00)	25% (0.01–0.49)

**Table 2C:** Comparison of Tau/GFAP combination receiver operating characteristic (ROC) curve with Tau-ROC and GFAP-ROC.

**With**	***z*-value**	***p*-value**
ROC-Tau	0.25	0.80
ROC-GFAP	−0.23	0.81

### Biomarker Comparison Between Subjects With and Without Post-concussive Symptoms

**Figure 3:**
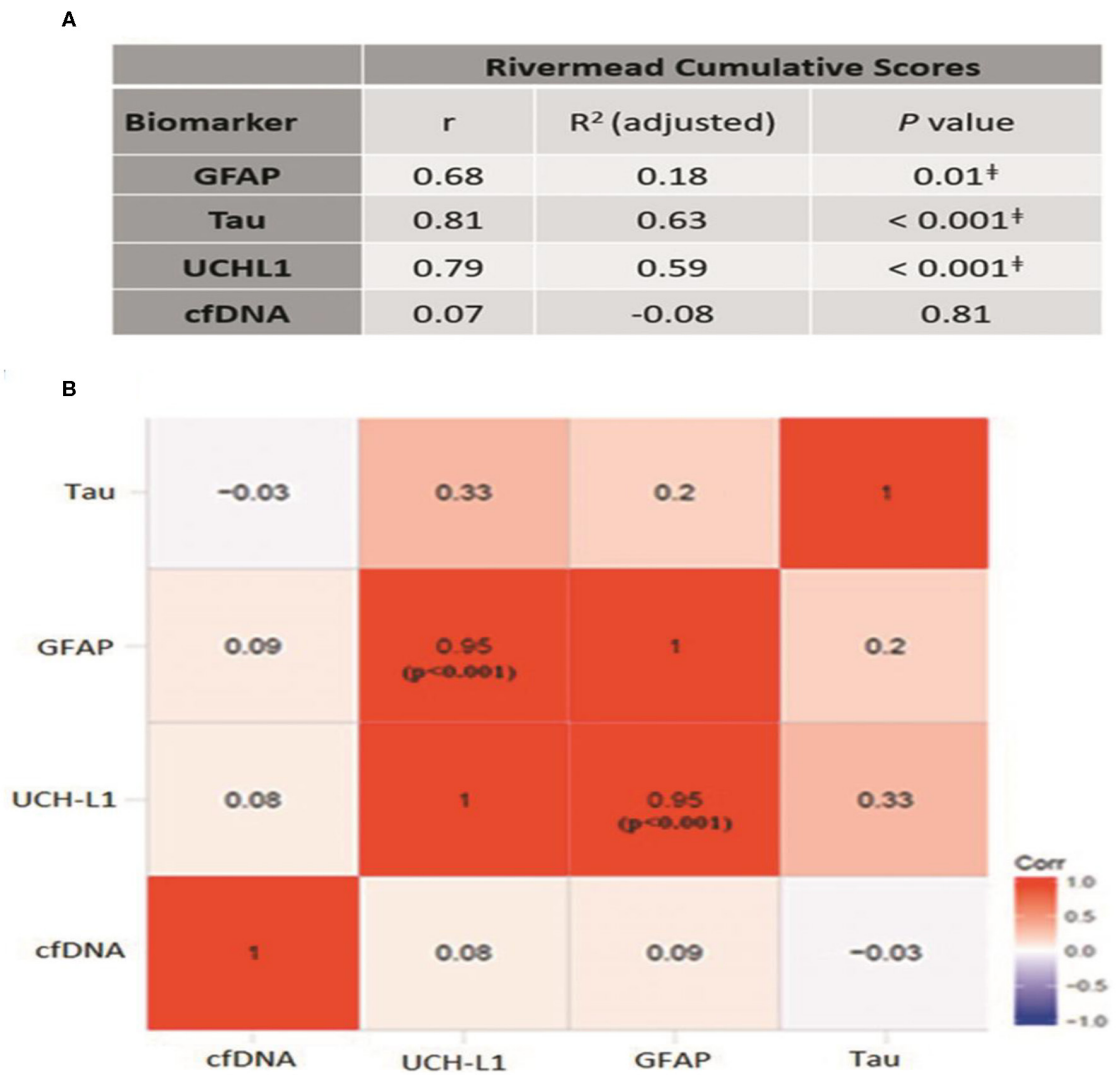
**(A)** Correlation between different biomarkers and cumulative Rivermead symptom scores; *p* ≤ 0.01 **(B)** Heat-map demonstrating the correlation between different biomarkers. Pearson correlation coefficient, r is mentioned in each box.

We next determined whether the presence of post-concussive symptoms is associated with elevated plasma GFAP, UCH-L1, Tau, or cfDNA. To this end, the Rivermead Post Concussion Symptoms Questionnaire (RPCSQ), a validated instrument for assessment of post-concussive symptoms following mild TBI (26, 27), was administered to study subjects by a trained study coordinator at 1-month follow-up. RPCSQ score was obtained for all 27 minor-TBI subjects. The highest cumulative RPCSQ scores were observed in the two patients with CT imaging abnormalities. No significant correlation was observed between the cumulative RPCSQ and cfDNA. However, we observed significant, positive correlation between plasma GFAP, UCH-L1 and Tau, and cumulative RPCSQ (Figure 3A). Specifically, the higher RPCSQ scores were associated with higher plasma biomarkers. The Pearson correlation between GFAP, UCH-L1, and Tau and cumulative RPCSQ were 0.68, 0.79, and 0.81, respectively (all *p* ɤ 0.01). In a correlation matrix analysis, we found that plasma GFAP and UCH-L1 levels were highly correlated (*r* = 0.95, *p* < 0.001) (Figure 3B). These results suggest plasma GFAP, UCH-L1, and Tau may be useful as predictive biomarker of post-concussive syndrome.

**Figure 4:**
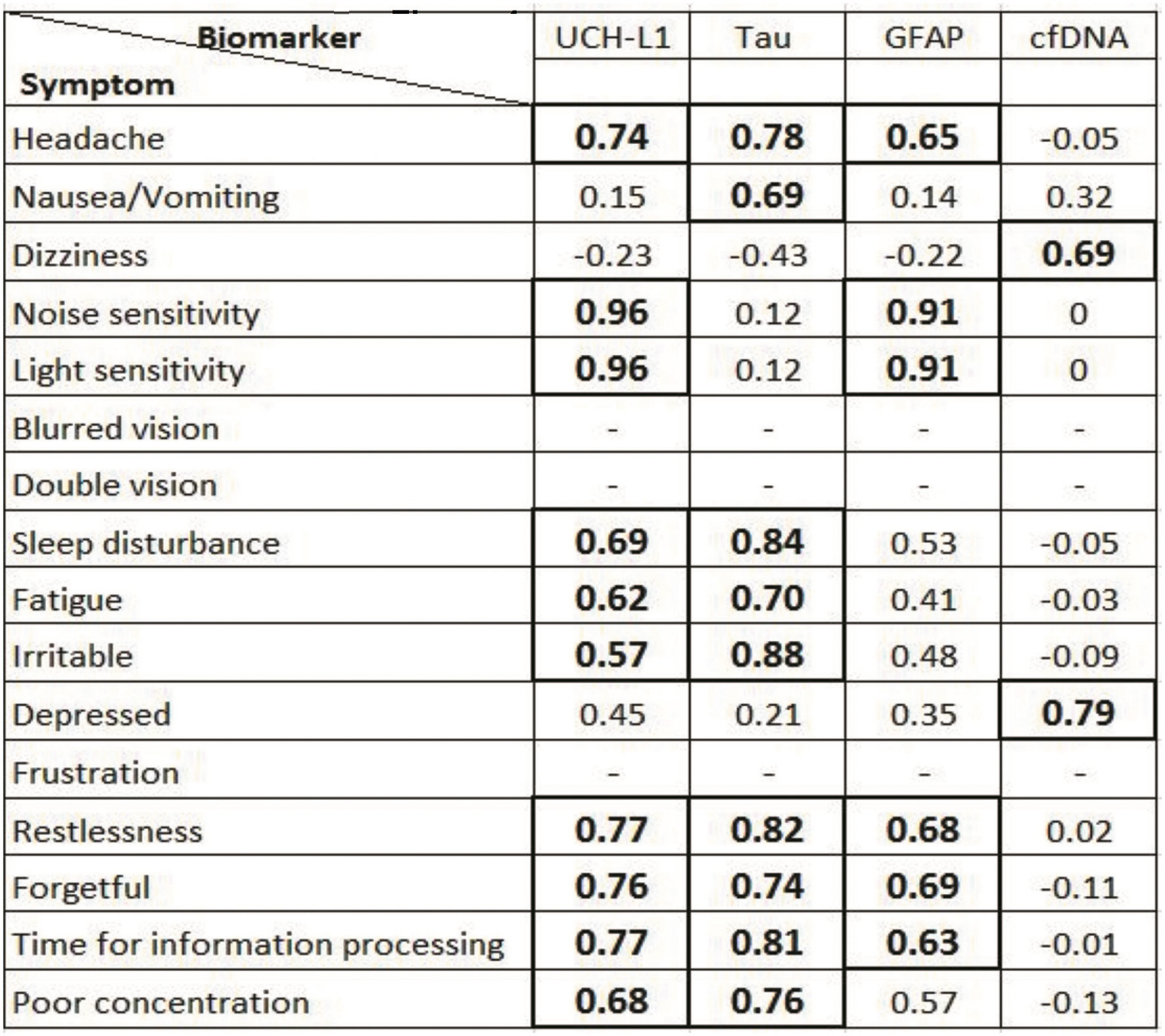
Correlation between different traumatic brain injury (TBI) biomarkers and Rivermead Questionnaire symptoms. Statistically significant (*p* < 0.05) Pearson co-efficient (r) is mentioned in bold format in box—represents symptoms that were not recorded in minor TBI patients.

Pertaining to each of the individual items on the RPCSQ, Plasma UCH-L1, and GFAP exhibited the highest correlation to sensitivity to noise and light (*r* = 0.96 and 0.91, respectively, both *p* < 0.001). Plasma UCH-L1 and Tau showed highest correlation with headache (*r* = 0.74 and 0.78, respectively, both *p* < 0.001), sleep disturbance (*r* = 0.69 and 0.84, respectively, both *p* < 0.001), and cognitive symptoms, including forgetfulness (*r* = 0.76 and 0.74, respectively, both *p* < 0.001), poor concentration (*r* = 0.68 and 0.76, respectively, both *p* < 0.001), and time required for information processing (*r* = 0.77 and 0.81, respectively, both *p* < 0.001). In contrast, cfDNA exhibited a strong correlation with depression (*r* = 0.79, *p* < 0.01) and dizziness (*r* = 0.69, *p* < 0.01) (Figure 4).

## Discussion

In this proof-of-principle study, we determined whether plasma isolated from minor TBI patients can be analyzed using an ACE-microarray platform (28). Because this platform had not been previously tested in this context, we selected TBI biomarkers with substantial literature supporting their utility in the study of TBI, including GFAP, UCH-L1, and Tau. We additionally tested whether cfDNA may serve as a useful biomarker. Our pilot data with the ACE-microarray platform support the utility of GFAP, UCH-L1, and Tau as plasma biomarker for TBI. Despite inherent constraints associated with the limited sample size, our pilot data are largely consistent with the previously published studies, including TRACK-TBI studies (37, 38). As such, we believe our data support ACE-microarray as a platform for blood-based biomarker study in patients suffering from minor TBI.

In comparison to the currently available methods of plasma analysis, the ACE micro-array platform presents several major advantages. First, ~25 μl of plasma is required, in comparison to other analytic platforms that require larger volumes. It is important to note that the entirety of this study was performed using blood left-over from standard chemistry tests. As such, if validated, the ACE micro-array platform may be added to the standard chemistry set without additional blood collection. Additionally, the ACE-microarray platform minimizes the number of steps in terms of sample transfer, and thereby reduces the risk for cross-contamination or sample mix-up. The only sample transfer step in the ACE-microarray platform was loading of the plasma onto the chip. In contrast, serial dilution of samples is typically required for sandwich enzyme-linked immunosorbent assays (ELISA). Finally, the chip can be subjected to multiplex immuno-fluorescent study to simultaneously assess biomarkers beyond Tau, GFAP, and UCH-L1.

A particularly intriguing result in this study involves the correlation between sub-domains of the RPCSQ and selected plasma biomarkers. Our results indicate that plasma UCH-L1, GFAP, Tau, and cfDNA levels correlated with different symptoms in the physical RPCSQ cluster domain. As a pilot analysis, the results should not be considered without scrutiny. For instance, meaningful quantitative assessment of this correlation is possible only in the context of the prevalence of the symptoms in the minor TBI population. Nevertheless, if the correlation reported here is validated in a future study, these results would suggest that the different aspects of post-concussive syndrome arise from pathophysiologic processes that ultimately lead to the release of the distinct biomarkers. A corollary of this hypothesis would suggest that medication that mitigates select patho-physiologic processes may be helpful to prevent or arrest post-concussive symptoms that compromise the patient's quality of life.

Admittedly, the predictive utility of the platform is sub-optimal given the data presented. Improvement in study design is warranted for future studies. For instance, our study did not factor into consideration- factors that affect serum concentration of GFAP, Tau, and UCH-L1, including extracranial injuries, neurological co-morbidities, and pre-injury functional status. Total Tau was tested in this study, which represents another limitation in this pilot analysis. Use of more specific and improved biomarkers like hyperphosphorylated Tau can improve the outcome prediction of TBI.

Additionally, given variability in the half-lives of these biomarkers, collection within 72 h of blood draw may present systematic bias in the correlative analyses. The reporting of RPCSQ at the time of follow-up is subject to recall bias. These and other potential confounding factors need to be considered in the design of future studies.

While this proof-of-principle study is, by definition, limited in its sample size and pilot in nature, the general demographic of the study population largely mirrors those of larger series, including the demographic of the study population and the proportion of minor TBI patients with abnormal head CT (39). The recapitulation of the association of GFAP, UCH-L1, and Tau with various aspects of TBI reported elsewhere is also reassuring (40). That said, we caution against definitive conclusions from this study beyond feasibility of ACE microarray as a potential biomarker platform. Studies reporting test-retest reliability of this assay are warranted in future. There are many steps ahead in clinical translation, including direct comparison of results derived from ACE-microarray platform with other established assays, such as the Quanterix assay (41). Prospective age and gender matched cohorts (38, 42) as well as thoughtful consideration of extracranial injuries, neurological comorbidities, and pre-injury functional status will be needed in advancing this biomarker platform toward clinical application.

## Conclusion

Our proof-of-principle study provides pilot data supporting the feasibility of the ACE microarray platform for plasma biomarker analysis in TBI patients.

## Data Availability Statement

The raw data supporting the conclusions of this article will be made available by the authors, without undue reservation.

## Ethics Statement

The studies involving human participants were reviewed and approved by Institutional Review Board, University of California San Diego. The patients/participants provided their written informed consent to participate in this study.

## Author Contributions

JL, MH, and CC conceived the study. JL and CC were responsible for collection of clinical specimens. JL and JA performed laboratory analysis of clinical specimens. SD analyzed the data. SD, JL, and CC wrote the manuscript. SD, AO, BS, and CC interpreted the findings. All authors contributed to the study design, advised on data analysis, edited the manuscript for intellectual content, and provided final approval of the manuscript. All authors contributed to the article and approved the submitted version.

## Conflict of Interest

MH declares the following competing financial interest: MH was a member of the Scientific Advisory Board for Biological Dynamics. The remaining authors declare that the research was conducted in the absence of any commercial or financial relationships that could be construed as a potential conflict of interest.
